# Construction of a prognostic assessment model for colon cancer patients based on immune-related genes and exploration of related immune characteristics

**DOI:** 10.3389/fcell.2022.993580

**Published:** 2022-12-16

**Authors:** Yanhua Wan, Yingcheng He, Qijun Yang, Yunqi Cheng, Yuqiu Li, Xue Zhang, Wenyige Zhang, Hua Dai, Yanqing Yu, Taiyuan Li, Zhenfang Xiong, Hongping Wan

**Affiliations:** ^1^ Department of Gastrointestinal Surgery, The First Affiliated Hospital of Nanchang University, Nanchang, China; ^2^ Department of General Surgery, The First People’s Hospital of Jiujiang, Jiujiang, China; ^3^ Queen Mary College of Nanchang University, Nanchang, China; ^4^ Department of Pathology, The First Affiliated Hospital of Nanchang University, Nanchang, China

**Keywords:** colon cancer, immune-related gene, immune cell infiltration, immune escape, risk model

## Abstract

**Objectives:** To establish a novel risk score model that could predict the survival and immune response of patients with colon cancer.

**Methods:** We used The Cancer Genome Atlas (TCGA) database to get mRNA expression profile data, corresponding clinical information and somatic mutation data of patients with colon cancer. Limma R software package and univariate Cox regression were performed to screen out immune-related prognostic genes. GO (Gene ontology) and KEGG (Kyoto Encyclopedia of Genes and Genomes) were used for gene function enrichment analysis. The risk scoring model was established by Lasso regression and multivariate Cox regression. CIBERSORT was conducted to estimate 22 types of tumor-infiltrating immune cells and immune cell functions in tumors. Correlation analysis was used to demonstrate the relationship between the risk score and immune escape potential.

**Results:** 679 immune-related genes were selected from 7846 differentially expressed genes (DEGs). GO and KEGG analysis found that immune-related DEGs were mainly enriched in immune response, complement activation, cytokine-cytokine receptor interaction and so on. Finally, we established a 3 immune-related genes risk scoring model, which was the accurate independent predictor of overall survival (OS) in colon cancer. Correlation analysis indicated that there were significant differences in T cell exclusion potential in low-risk and high-risk groups.

**Conclusion:** The immune-related gene risk scoring model could contribute to predicting the clinical outcome of patients with colon cancer.

## Introduction

Colon cancer is a common malignant tumor that arises from cells in the colon of the gastrointestinal tract (Hofseth et al., 2020). Globally, there are more than 1 million new cases of colon cancer each year, accounting for about 10% of all new cancer cases (O'Keefe, 2016). Despite advances in surgery, chemotherapy, and radiation over the past few decades, the prognosis for patients with advanced and metastatic colon cancer remains poor (Okugawa et al., 2015; Siegel et al., 2017). Therefore, it is urgent to explore the pathogenesis and development mechanism of colon cancer, find effective biomarkers to predict the prognosis of patients and explore potential therapeutic targets.

With the in-depth study of molecular biology and immune mechanism, the rapid development of cancer immunotherapy has brought more possibilities for cancer treatment in recent years (Wang et al., 2021). It has been proved that monoclonal antibody blocking cytotoxic T-lymphocyte-associated antigen 4 (CTLA-4) and programmed cell death protein 1 (PD-1) immune checkpoint pathways and chimeric antigen receptor T cell therapy have achieved initial success in clinical treatment of cancer patients (Yang, 2015). However, immunotherapy only works effectively in a minority of cancer patients (Dai et al., 2021). With the emergence of immunosuppressive microenvironment and the high-frequency mutation of tumor cells, tumors can develop resistance to immunotherapy through various escape mechanisms (Beatty and Gladney, 2015). Thus, screening out patients who respond best to immunotherapy remains a challenging clinical issue. The increasing number of research reveals that immune-related genes can be used to diagnose cancer stage and prognosis. Immune-related gene pairs have been proved to become effective biomarkers for predicting the prognosis of gastric, breast and bladder cancer patients, providing assistance in identifying immunotherapy response of patients (Mu et al., 2021; Xiong et al., 2021; Zhao et al., 2021). Although several prognostic markers of colon cancer based on immune-related genes have been established, there is a lack of a more comprehensive indicator to predict patient survival and immunotherapy response. Thus, by exploring the relationship between immune-related genes and survival, immune cells and immune escape in patients with colon cancer, we can provide support for patient immunotherapy.

In this study, we used machine learning to analyze the expression of 679 immune-related genes and their relationship with prognosis and immunotherapy response in 451 patients with colon cancer. A novel prognostic risk model of immune-associated genes was developed to predict overall survival in colon cancer patients. In addition, we focused on the relationship between risk score of model and tumor-infiltrating immune cells, immune function, and immune escape. By assessing the immune characteristics of patients in the high-risk and low-risk groups, we could predict the response of colon cancer patients to immunotherapy.

## Materials and methods

### Research object

We downloaded the mRNA expression profile data, corresponding clinical information and somatic mutation data of patients with colon cancer from the Cancer Genome Atlas (TCGA) database (https://portal.gdc.cancer.gov/). The sample included 454 patients with colon cancer. The gene expression data of the GSE40967 dataset was downloaded from the GEO database as the test set. We used the Maftools R software package for visualized analysis of somatic mutation data.

### Differential gene analysis

Differential expression data were analyzed by limma R software package. *p* < 0.05 and logFC>1 were regarded as the standard to screen out differentially expressed genes (DEGs).

### Gene functional enrichment analysis

ClusterProfiler function package in R was used for GO (Gene ontology) and KEGG (Kyoto Encyclopedia of Genes and Genomes) enrichment analysis of immune-related genes. *p* < 0.05 was considered as significant enrichment.

### Weighted gene co-expression network analysis

We used WGCNA R package to analyze the co-expression of immune-related genes in colon cancer. The analyses of scale-free topological models and average connectivity for various soft threshold powers were performed. WGCNA algorithm was used to screen out co-expressed gene modules, and then the correlation between these modules and sample phenotypes was analyzed. Modules were selected for further analysis based on the number of immune genes aggregated and the differences between cancer and normal tissues.

### Immune-related gene survival analysis

The RNA-seq data of immune-related genes in colon cancer patients wascombined with corresponding clinical data. The relationship between survival time of colon cancer patients and immune-related gene expression was evaluated by univariate Cox regression analysis. The genes selected by COX regression were divided into high and low expression groups according to gene expression levels. The Kaplan-Meier method was used to estimate overall survival in high expression and low expression groups. *p* <0.05 was statistically significant.

### Establishment of immune-related gene risk model

Based on the TCGA dataset of colon cancer patients, LASSO regression (estimated penalty parameters using 10-fold cross validation) and multivariate Cox regression were used to establish risk models associated with immune genes. We calculated the risk scores of patients according to the expression of the model genes and the formula is Risk score = (0.2768)*AEN + (-0.2698)*LGALS4 + (-0.0915)*XDH. After risk calculation, we divided the sample into high-risk and low-risk groups by median. Log rank was used to test the Kaplan-Meier survival analysis to compare survival differences between the two or more groups mentioned above. Time ROC analysis was performed to compare the prediction accuracy and risk score of immune-related genes. R software package glmnet was used for the above analysis. We used the Log-rank test and univariate Cox proportional hazard regression to obtain Kaplan-Meier curves, *p* values and hazard ratios (HR) with 95% confidence intervals (CI). The gene expression data of colon cancer patients in the GSE40967 dataset were used as the test set to verify the model. Multivariate Cox regression analysis was used to analyze the independence of risk score. Gene set enrichment analysis (GSEA) was conducted to analyze gene-enriched signaling pathways in the high-risk and low-risk groups. We used R package to display the mutation types of the high frequency mutant genes in the high-risk and low-risk groups in the waterfall diagram. *p* < 0.05 was considered as statistically significant.

### Evaluation of tumor immune cell infiltration and immune functions in colon cancer

We divided patients into high and low-risk groups according to the median risk score. Bioinformatics algorithm CIBERSORT was used to estimate 22 types of tumor-infiltrating immune cells in tumors by characterizing the cellular composition of complex tissues based on standardized gene expression profiles. We used R package to visualize the condition of 22 types of tumor-infiltrating immune cells in each colon cancer patient in the high-risk and low-risk groups. The differences of immune cell infiltration in different risk groups were analyzed by limma and ggplot package. The Kaplan-Meier method was used to estimate overall survival in different levels of immune cells. We used limma, GSVA and GSEABase R package to analyze the relationship between immune function and risk score. The boxplot was created by R package to present differences in immune function between high-risk and low-risk groups. The Kaplan-Meier method was conducted to estimate overall survival in different levels of immune functions. For each immune cell type or function, we also calculated the median express level according to the expression level of the cell or function in all patients. Those with lower expression level than the median were considered as the low expression group, while those with higher expression level were considered as the high expression group.

### Analysis of immune escape and clinical characteristics in different risk groups

Linear regression analysis was conducted to evaluate the correlation between risk score and CD274 gene expression. The ggpubr R package was used to visualize the results in the form of scatter plot. The difference in CD274 gene expression in the high-risk and low-risk groups was shown in the boxplot created by R package. We used linear regression analysis to evaluate the correlation between the risk score and tumor mutation burden (TMB). The ggpubr R package was used to visualize the results in the form of scatter plot. The difference in TMB in the high-risk and low-risk groups was shown in the boxplot created by R package. ComplexHeatmap R package was performed to analyze the differences in clinical characteristics of colon cancer patients in the high-risk and low-risk groups, and was used to plot the heatmap. We further used Rcolorbrewer to numerically display the differences of age in the high and low immune-related gene prognostic index (IRGPI) groups in the quadrangle map. The difference of immunophenotype in the high and low IRGPI groups in the quadrangle map was numerically presented by Rcolorbrewer. We evaluated differences in immune escape between high-risk and low-risk groups. The ggplot R package was used to visualize the results.

### Experimental validation of immune-related prognostic genes

From September 2017 to September 2020, 60 patients with stage I and III colon cancer in the First Affiliated Hospital of Nanchang University were screened. Inclusion criteria: (Hofseth et al., 2020) Pathologically confirmed cases (O'Keefe, 2016) New cases first diagnosed in this hospital. Exclusion criteria: (Hofseth et al., 2020) With hypertension systemic diseases such as cardiovascular and cerebrovascular diseases, diabetes or other malignant tumors (O'Keefe, 2016) Patients receiving preoperative radiotherapy, chemotherapy or other antitumor drugs. Tumor tissue and normal tissue samples were collected from the surgical patients (tissues further than 5 cm from the periphery of tumor tissue were regarded as normal tissues). The basic information of patients with stage I and stage III colon cancer was shown in Table 1. To further confirm the expression of key genes in COAD, we performed immunohistochemical (IHC) staining. Collected 40 samples of COAD patients, including 20 stages I and III respectively. The samples were embedded in paraffin and incubated with first antibody at 4°C overnight. Then, a secondary antibody coupled to horseradish peroxidase (HRP) was incubated with the sections for 60 min at room temperature, followed by 3,3’ -diaminobenzidine (DAB substrate system; 2005289) and hematoxylin staining. Images were taken with an Olympus cx-21 (Japan) magnified ×200. Using the analysis software Image-Pro Plus 6.0 (Media Cybernetics, Inc., Rockville, MD, United States), the same intensity of brown color was selected as the unified standard to judge the positivity of all photos, and each photo was analyzed to obtain the positive cumulative optical density (IOD) and tissue pixel area (AREA). The average optical density IOD/AREA (mean density) was calculated. SPSS 19.0 software was used for independent sample t-tests of the average optical density values obtained from the stages I and III of COAD tissues. *p* < 0.05 was defined as statistically significant.

**TABLE 1:** Comparison of baseline data of patients with stage I and stage III colon cancer.

Group/Character	Age	Male	Female	Pathological type
stage I	62.6 ± 2.5	25	35	Adenocarcinoma
stage III	64.3 ± 1.5	28	32	Adenocarcinoma
*p* value	>0.05			>0.05

### Single-cell data analysis

The gene expression profiling data of the single-cell dataset GSE81861 was downloaded from the GEO database; the total gene expression levels of the target genes in all cells of colorectal cancer tissue and normal tissue were extracted for comparison. Data quality control was performed on the single-cell data, and epithelial cells were selected; the expression differences of target genes in cancer cells and normal intestinal epithelial cells were analyzed and compared.

### Statistical analysis

R software version 4.0.3 (The R Foundation for Statistical Computing, 2020) was conducted for statistical analysis. The Kaplan-Meier method was used to determine the overall survival rate in each group. Log-rank was conducted to analyze the significant difference in survival rate among different groups. Wilcoxon sign rank sum test was used to analyze the comparison of immune cell infiltration in each group. *p* <0.05 was considered statistically significant.

## Result

### Analysis of differentially expressed immune-related genes

**FIGURE 1:**
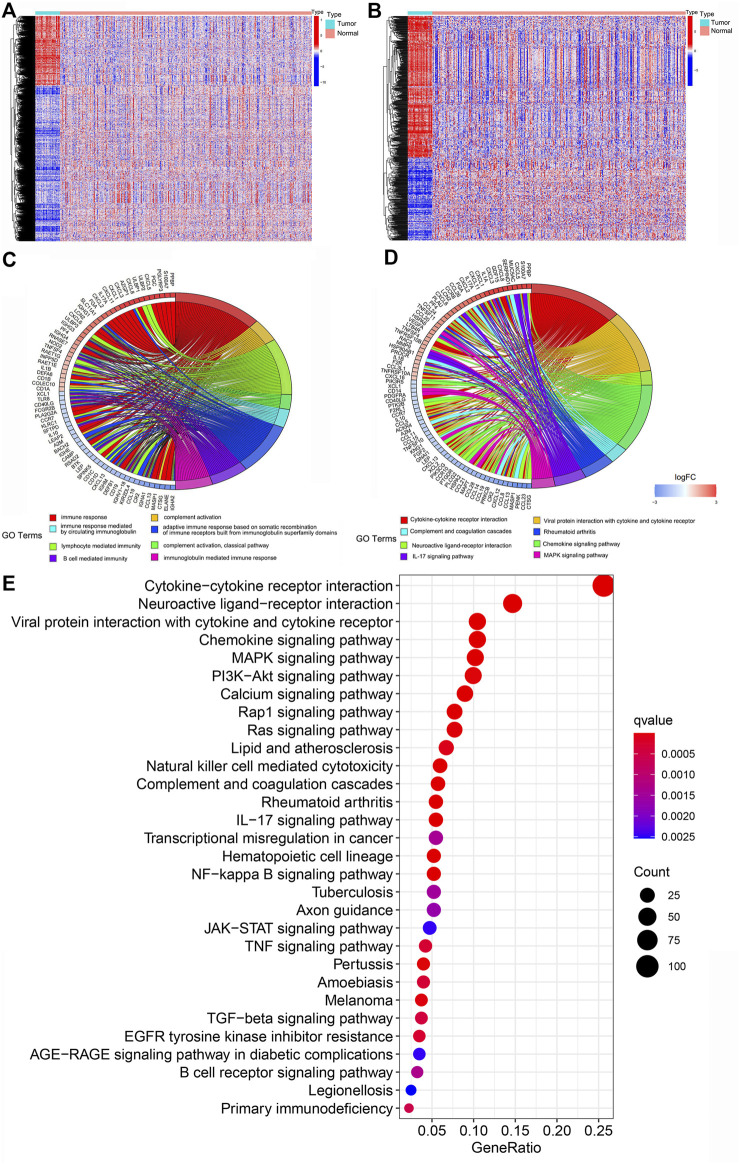
Results of differential gene analysis and enrichment analysis. **(A)** The heat map of the differentially expressed genes (DEGs). **(B)** The heat map of the immune-related DEGs. The horizontal axis is the sample. The vertical axis is different genes. Red indicated the high expression of the gene. Blue indicated the low expression of the gene. **(C)** The Circos plot of the main GO term for immune-related DEGs enrichment. **(D)** The Circos plot of the main KEGG signaling pathways for immune-related DEGs enrichment. **(E)** The bubble plot of the main KEGG pathways. The horizontal axis of the map indicated the gene ratio. The longitudinal axis indicated the names of each pathway.

In 451 patients with colon cancer, 679 differentially expressed genes (DEGs) associated with immune reactions were identified by comparing cancer samples with normal tissues. The heatmaps revealed the difference in the expression of DEGs and immune-related DEGs between cancer and para-cancer samples (Figures 1A,B).

### Functional enrichment analysis of immune-related genes

To investigate the DEG-associated biological processes and signaling pathways, functional enrichment analysis of immune-related DEGs was performed. We found that 679 genes were significantly enriched in GO pathways, including immune response, complement activation and so on (Figure 1C). Results of KEGG showed that these genes are mainly enriched in cytokine-cytokine receptor interaction and other KEGG pathways (Figures 1D,E).

### Construction of weighted gene co-expression network analysis co-expression model

**FIGURE 2:**
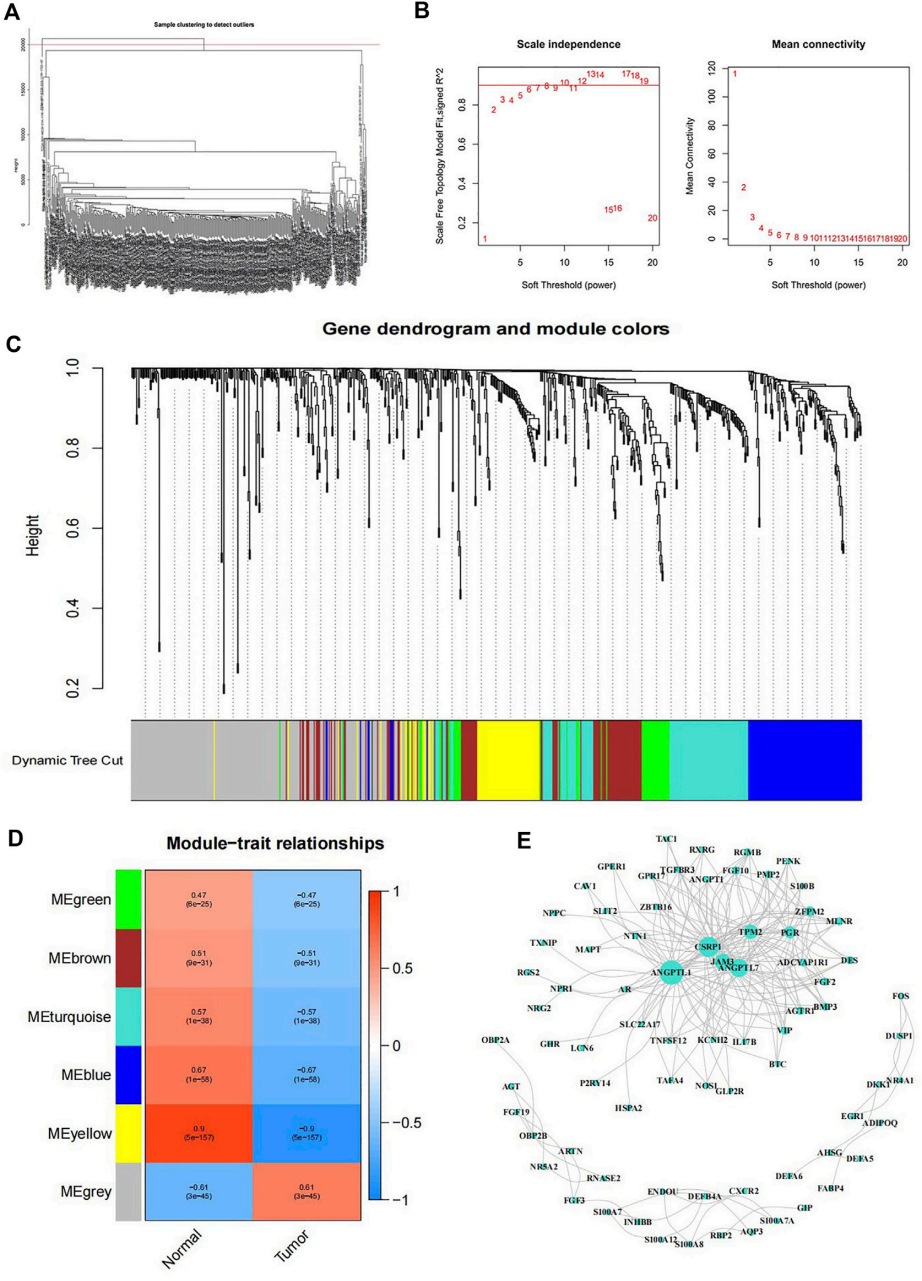
WGCNA co-expression model **(A)** The tree diagram of gene cluster analysis. **(B)** Left: Analysis of the scale free topology model fit for various soft threshold powers. Red line indicated Scale Free Topology Model Fit, signed R^2^ was 0.90. Right: Mean connectivity analysis of various soft threshold powers. **(C)** Gene dendrogram and module colors. **(D)** Heatmap of the correlation between modules and samples traits. The numbers in each square indicated the Pearson correlation coefficient (up) and *p* value (down). **(E)** Interaction network of genes in the grey module.

To establish a co-expression network, WGCNA was conducted to cluster samples and analyze the immune-related DEGs. The division and unsupervised clustering of all samples was showed in Figure 2A and the WGCNA model parameters were indicated in Figure 2B. According to the gene expression level, the co-expression correlation network of genes is calculated and these genes can be divided into different clusters indicated by distinctive colors (Figure 2C). To identify the tumor immunity related module, we used a correlation analysis between modules and traits (Figure 2D). According to the number of clustered immune genes and the difference between cancer and normal tissues, we selected the grey module with the largest number of genes for network establishment, and the result was shown in Figure 2E.

### Identification of prognostic immune-related differentially expressed genes

**FIGURE 3:**
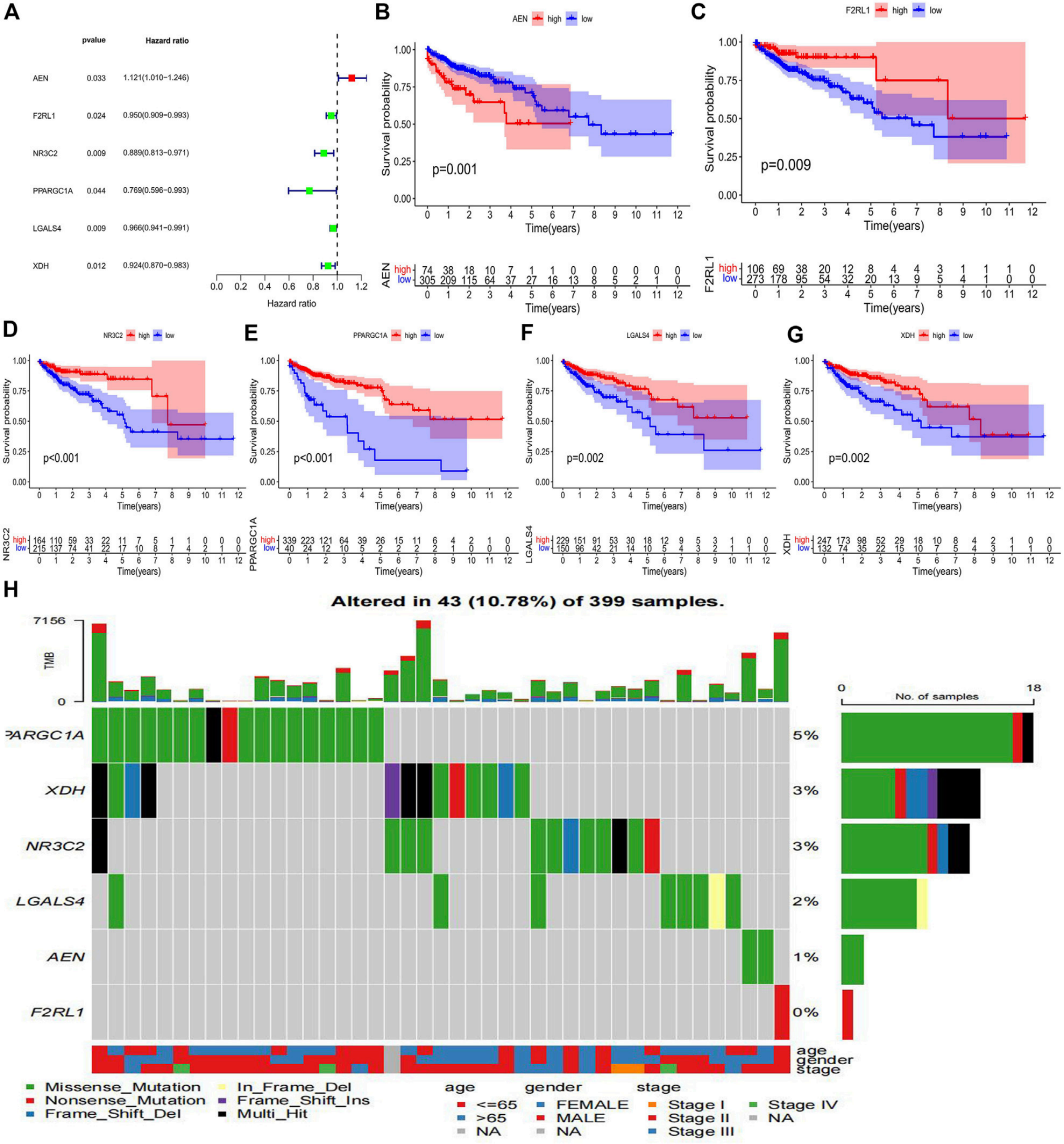
Identification of prognostic immune-related genes in colon cancer **(A)** The forest map of 6 immune-related genes significantly associated with colon cancer prognosis. **(B–G)** Kaplan Meier survival curves of colon cancer samples with different expression levels of 6 immune-related prognostic genes. **(H)** The waterfall plot of mutation types of prognostic genes in colon cancer patients.

To determine which genes can serve as independent prognostic factors, we performed univariate Cox regression analysis using the TCGA dataset to calculate Hazard ratio (HR) for each gene. *p* < 0.05 was used as the filtering threshold. Finally, six prognostic genes were identified, among which the AEN with HR values greater than 1 was a risk gene, while the F2RL1, NR3C2, PPARGC1A, LGALS4, and XDH with HR values less than 1 were protective genes (Figure 3A). The result of univariate Cox regression analysis was shown in Table 2. The mean value of the specific gene expression was calculated, which was used to divide the high and low-expression groups. Figures 3B–G showed the relationship between the expression of these 6 genes and the prognosis of patients with colon cancer. The mutation types of prognostic genes in 43 colon cancer patients were shown in Figure 3H.

**TABLE 2:** The univariate Cox regression analysis result of the six immune-related prognostic genes in colon cancer.

Gene	HR	HR.95L	HR.95H	*p* value
AEN	1.121447287	1.009530509	1.245771184	0.032614724
F2RL1	0.949993071	0.908564929	0.993310226	0.024132478
NR3C2	0.888641395	0.813399067	0.970843909	0.008910291
PPARGC1A	0.769165253	0.595788236	0.992995749	0.044020027
LGALS4	0.965719378	0.940723799	0.991379104	0.009131688
XDH	0.924413669	0.869665922	0.982607931	0.011628202

### Construction and validation of prognostic model in colon cancer patients

**FIGURE 4:**
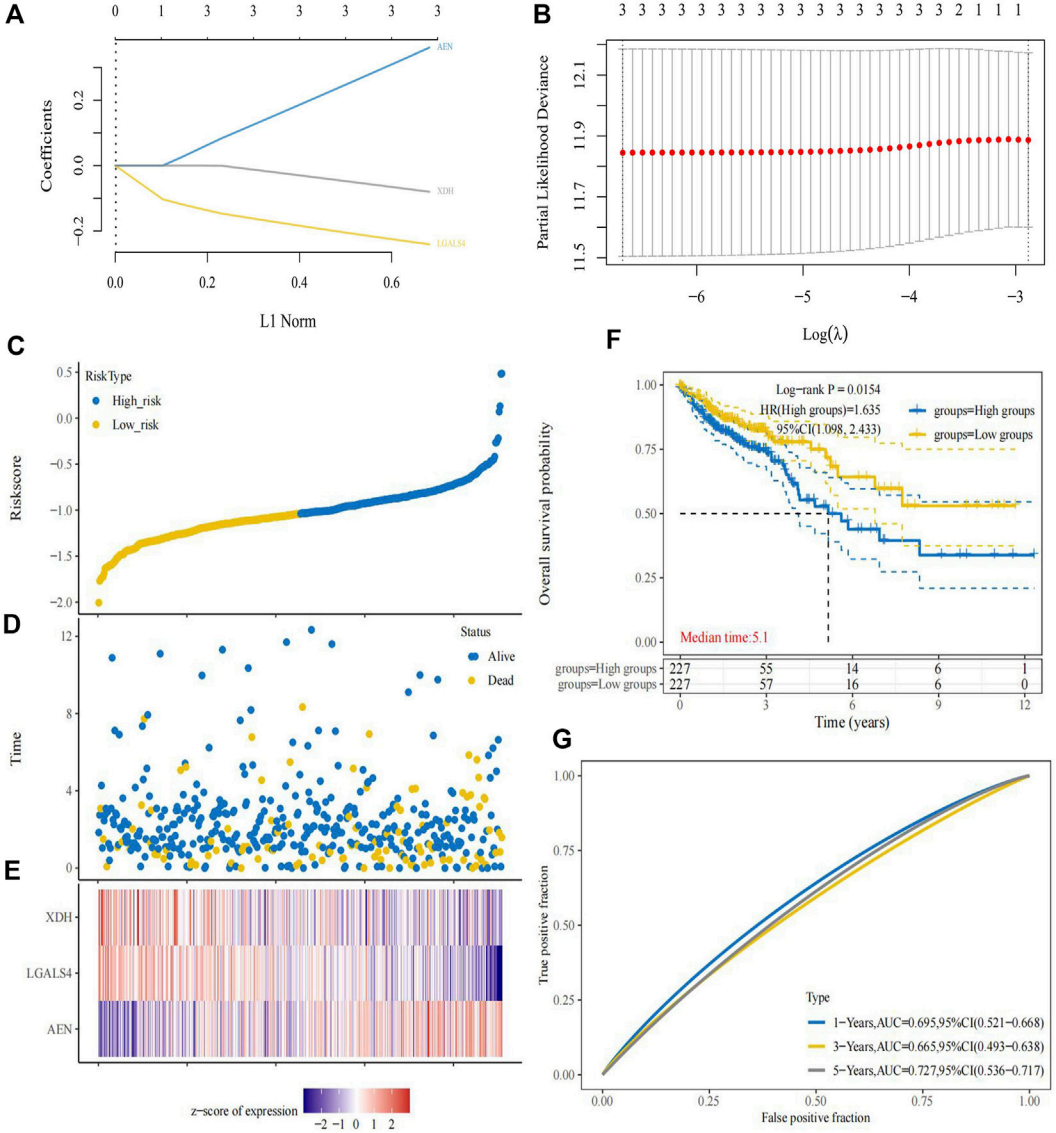
Establishment of prognostic risk model. **(A)** The coefficients of the selected features are shown by the lambda parameter, with the abscissa representing the value of the independent variable lambda and the ordinate representing the coefficient of the independent variable. **(B)** Partial likelihood deviations were plotted against log(λ) using the LASSO Cox regression model. **(C)** The risk score distribution between the low-risk and high-risk groups. **(D)** The survival status and survival time of patients in the low-risk and high-risk groups. **(E)** Risk-related heatmap of three immune-related genes in risk model. **(F)** The Kaplan-Meier survival curve of colon cancer samples in the low-risk and high-risk groups. **(G)** ROC curve of the prognostic signature to evaluate the accuracy.

**FIGURE 5:**
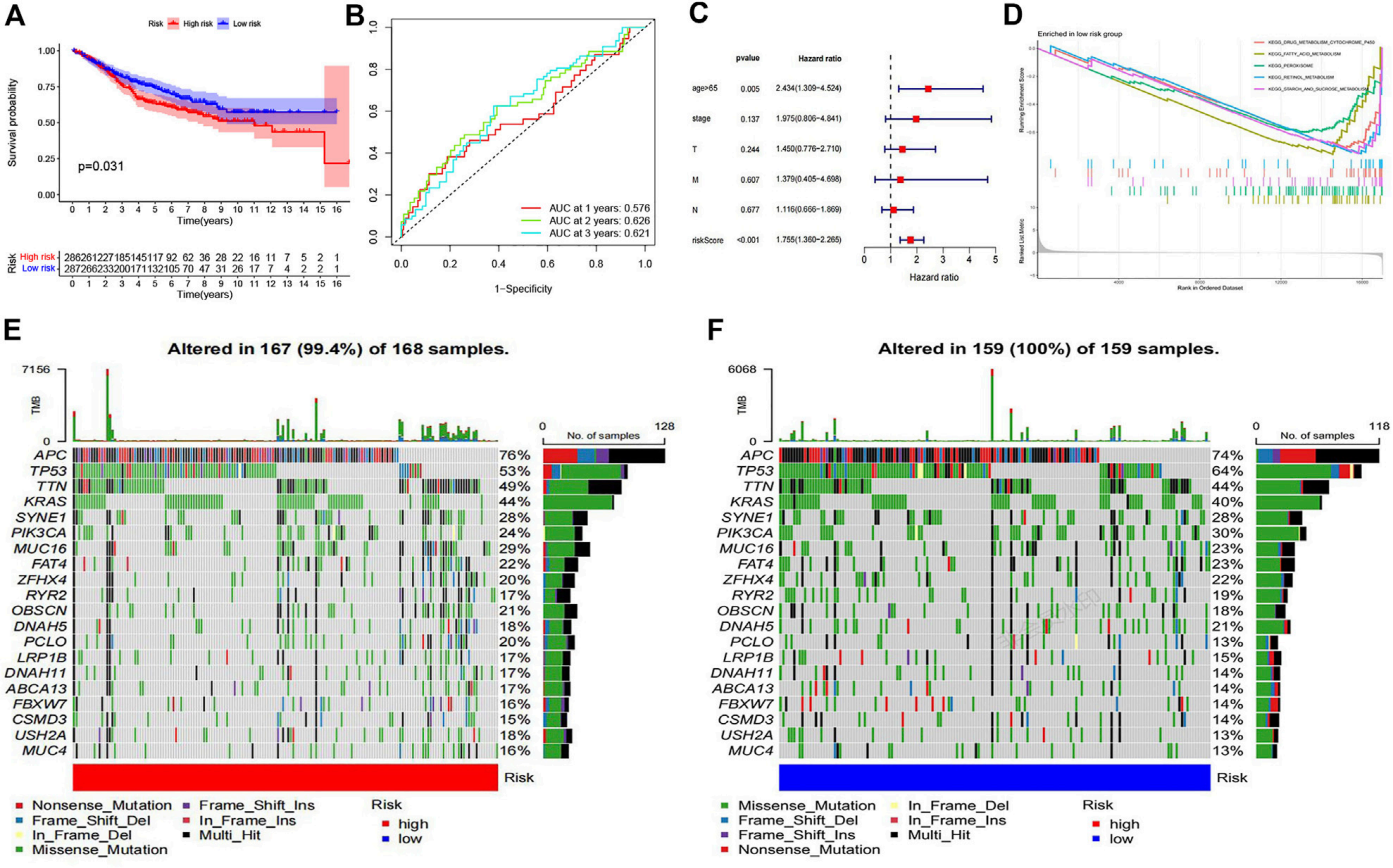
Verification of risk model and differential landscape of somatic mutation burden in the low-risk and high-risk groups **(A)** The Kaplan-Meier survival curve of colon cancer samples in GSE40967 dataset. **(B)** ROC curve of the test set. **(C)** The forest plot of multivariate cox regression analysis. **(D)** GSEA showed five pathways enriched in the low-risk group. **(E)** The waterfall plot of mutation details and tumor mutation burden (TMB) for each colon cancer patient sample in the high-risk group. **(F)** The waterfall plot of mutation details and tumor mutation burden for each colon cancer patient sample in the low-risk group.

According to the expression of prognostic genes and the regression coefficient of regression analysis, parameters for Lasso regression analysis model were built and a risk scoring model was established to predict the survival of patients (Figures 4A,B). According to the risk score formula, we used the expression of model genes to calculate the risk score which is thereby used for dividing the two risk groups. The risk score distribution between the low-risk and high-risk groups was shown in Figure 4C. The survival status and survival time of patients in different risk groups were shown in Figure 4D. Figure 4E revealed that the relative expression of 3 immune-related prognostic genes in each patient. Survival analysis showed that the overall survival of the high-risk group was worse than that of the low-risk group (*p* = 0.0154) (Figure 4F). In addition, the area under the curve (AUC) of 1-year overall survival was 0.695; 3-year overall survival was 0.665; 5-year overall survival was 0.727. (Figure 4G). For test set, we also divided patients into high and low-risk groups according to the model gene expression and the risk score formula. Kaplan-Meier survival analysis demonstrated that the survival of the low-risk group was better than that of the high-risk group in the GSE40967 data sets (Figure 5A). The AUC of 1-year overall survival was 0.576; 2-year overall survival was 0.626; 5-year overall survival was 0.621 (Figure 5B). Multivariate Cox analyses were used to evaluate whether the risk score was an independent prognostic factor in colon cancer patients. The hazard ratio (HR) of the risk score was 1.755 and 95% confidence interval (CI) was 1.360–2.265 (*p* < 0.001). These results revealed that risk score and age were significantly associated with overall survival (OS) in colon cancer. While stage, T, N, and M stage were uncorrelated with OS (Figure 5C). The higher the risk score, the higher death risk is a harmful prognostic indicator.

To further explore the role of immune-related genes in colon cancer biological processes and signaling pathways, we used GSEA to identify potentially regulatory mechanisms in the low-risk group. Results of enrichment analysis showed that drug metabolism cytochrome P450, fatty acid metabolism, peroxisome, retinol metabolism and starch and sucrose metabolism pathways were significantly enriched in the low-risk group (Figure 5D). Mutation details and tumor mutation burden for each colon cancer patient sample in the high-risk and low-risk groups were shown in waterfall diagrams (Figures 5E,F). We were able to compare the different types of mutations in high-frequency mutated genes in the high-risk and low-risk groups.

### Immunocyte characteristics between the low-risk group and high-risk group

**FIGURE 6:**
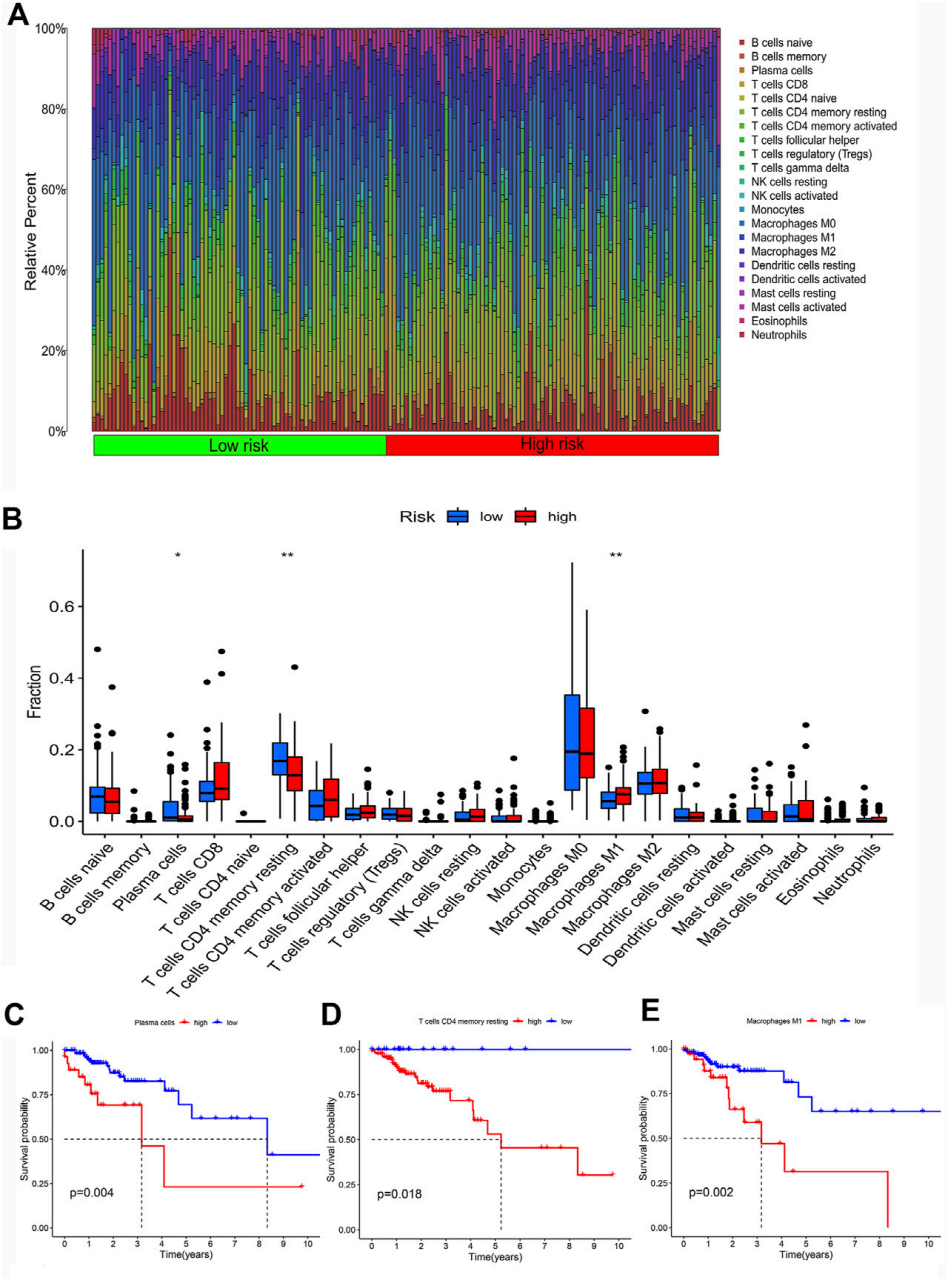
Infiltration characteristics of immune cells. **(A)** The bar plot of the percentage of 22 immune cell subtypes in each colon cancer sample. **(B)** The box plot of differences in infiltration of 22 types of immune cells in low-risk and high-risk groups. **(C–E)** The Kaplan-Meier survival curve of colon cancer patients with different levels of plasma cells, T cells CD4 memory resting and Macrophage M1.

To investigate the difference of immune features between high and low-risk groups, immune cell analysis was conducted. The bar chart presented the percentage of 22 immune cell subtypes in each colon cancer sample by different colors (Figure 6A). The Wilcoxon rank-sum test revealed that the proportion of plasma cells and T cells CD4 memory resting in the low-risk group was higher than that in the high-risk group, while the proportion of macrophage M1 in the low-risk group was lower than that in the high-risk group (Figure 6B). To explore the relationship between the proportion of immune cell infiltration and patient survival, survival analysis revealed that a high proportion of plasma cells, T cells CD4 memory resting and macrophage M1were significantly associated with poor prognosis in colon cancer patients (Figures 6C–E).

**FIGURE 7:**
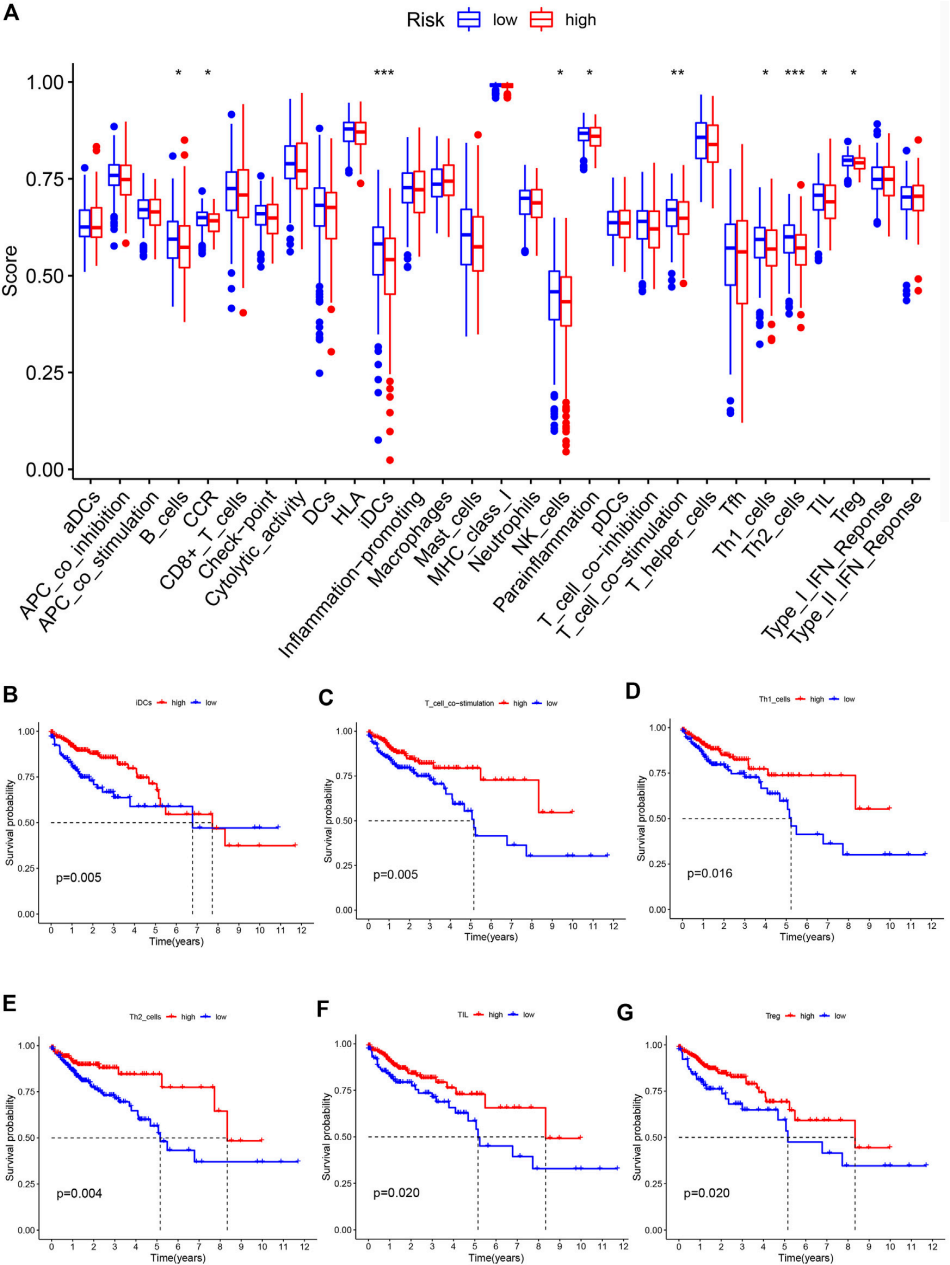
Characteristics of immune function in different risk groups **(A)** The box plot of risk scores of immune cells and immune functions in low-risk and high-risk groups. The overall survival rate of different levels of iDCs **(B)**, T cell co-stimulation **(C)**, Th1 cells **(D)**, Th2 cells **(E)**, TIL **(F)** and Treg **(G)**.

We analyzed differences in immune cell infiltration and immune function activation between the low-risk and high-risk groups. Results showed that B cells, iDCs, NK cells, Th1 cells, Th2 cells, TILs, and Treg cells were significantly reduced in the high-risk group. For immune-related functions, CCR, parainflammation and T-cell costimulation were enriched in low-risk group (Figure 7A). Survival analysis revealed that high levels of iDCs, T cell co-stimulation, Th1 cells, Th2 cells, TIL, and Treg were significantly associated with favorable prognosis (Figures 7B–G).

### Correlation analysis of the PD-L1 level, tumor mutation burden level and clinical characteristics in different risk groups

**FIGURE 8:**
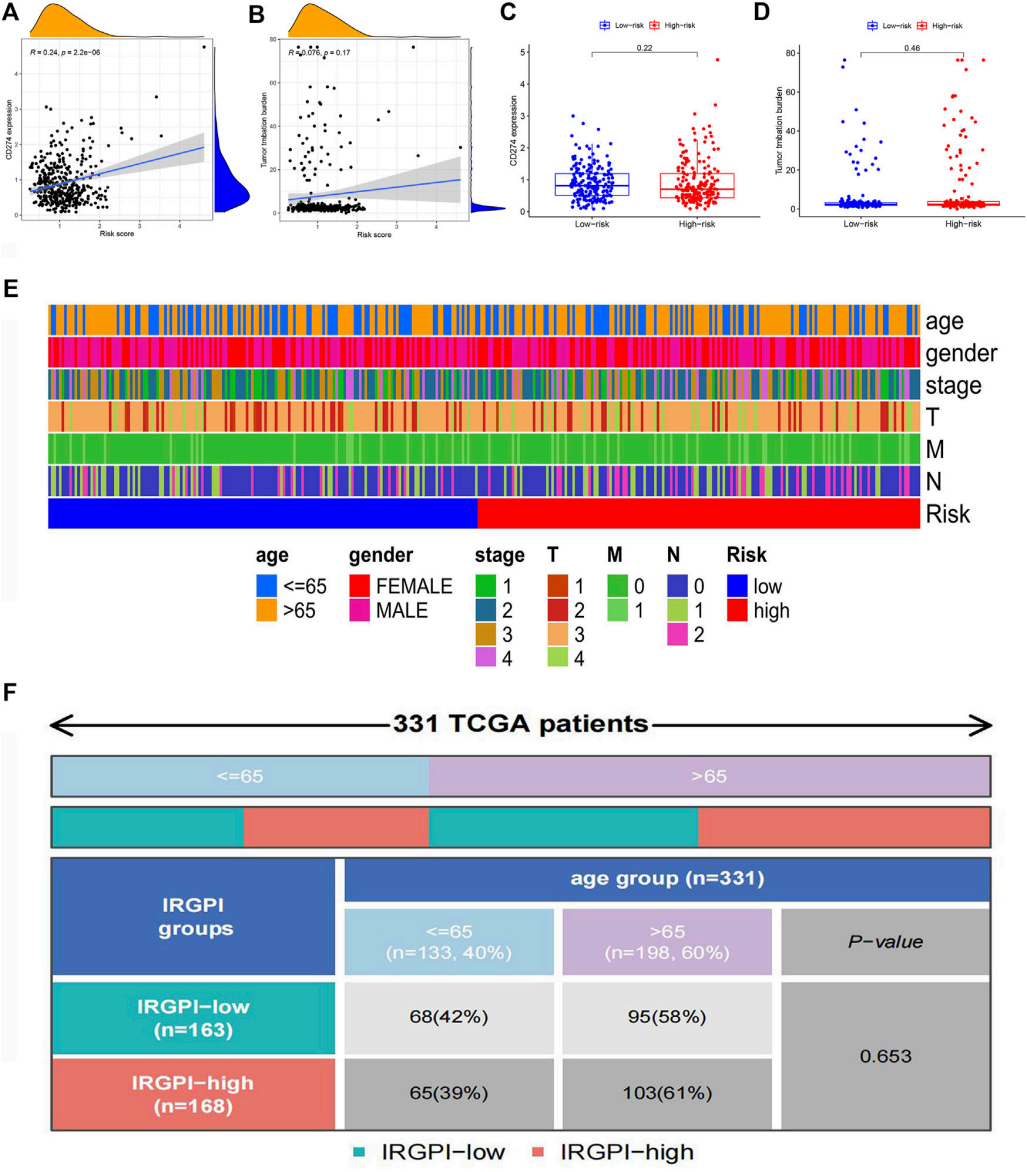
Correlation analysis of clinical characteristics in different risk groups **(A)** The scatterplot of correlation analysis of risk score and CD274 gene expression. **(B)** The scatterplot of correlation analysis of risk score and TMB. **(C)** The box plot of the analysis of differences in CD274 gene expression in low-risk and high-risk groups. **(D)** The box plot of the analysis of differences in TMB in low-risk and high-risk groups. **(E)** The heat map of clinical characteristics in low-risk and high-risk groups. **(F)** The quadrangle map of the analysis of differences in ages in IRGPI-low and IRGPI-high groups.

PD-L1 was one of the most popular immune checkpoints associated with cancers, including colon cancer (Masugi et al., 2017). Therefore, we analyzed the correlation between the risk score and PD-L1 level. As TMB is a hot topic in cancer research, we also analyzed the correlation between TMB and the risk score. PD-L1 expression is positively correlated with the risk score while no significant correlation is observed between TMB and the risk score (Figures 8A,B). There were no significant differences in PD-L1 expression and TMB between the low-risk group and the high-risk group (Figures 8C,D). Figure 8E showed that the differences in clinical features between the high-risk group and low-risk group. There were significant differences in the M stage between high-risk and low-risk patients. Clinical correlation analysis revealed that there were no significant differences in age in the IRGPI-low group and IRGPI-high group (Figure 8F).

### Correlation analysis of the immune escape potential in different risk groups

**FIGURE 9:**
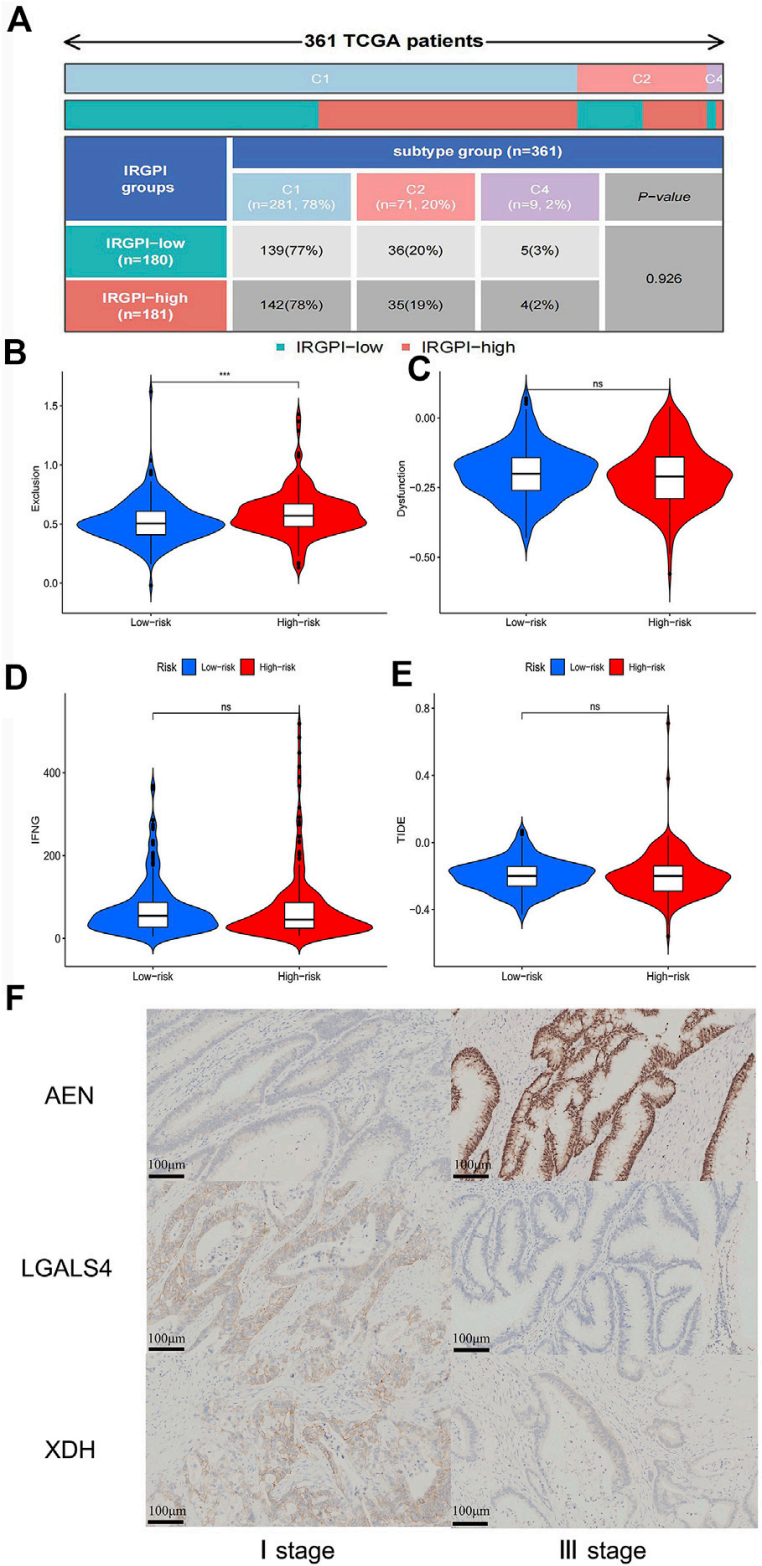
Correlation analysis of immune escape potential in different risk groups and immunohistochemical validation results **(A)** The quadrangle map of the analysis of differences in immune subtypes in IRGPI-low and IRGPI-high groups. **(B)** The violin plot of analysis of differences in T cells exclusion in low-risk and high-risk groups. **(C)** The violin plot of analysis of differences in T cells dysfunction in low-risk and high-risk groups. **(D)** The violin plot of analysis of differences in interferon gamma (IFNG) in low-risk and high-risk groups. **(E)** The violin plot of analysis of differences in Tumor Immune Dysfunction and Exclusion (TIDE) in low-risk and high-risk groups. **(F)** Immunohistochemical results of expression of AEN, LGALS4 and XDH in stage I and stage III colon cancer patients. *p* < 0.05.

Immune correlation analysis showed that immune subtypes were not associated with different IRGPI groups (Figure 9A). To explore the approach of immunotherapy in colon cancer patients, we analyzed the relationship between immune escape mechanism and risk score. Results demonstrated that T cell exclusion potential was significantly increased in the high-risk group compared to the low-risk group. There was no significant difference in T cell dysfunction, IFNG and TIDE in two groups (Figures 9B–E).

### Validation the expression of key genes from immunohistochemical staining

To further verify the reliability of the above results, we performed immunohistochemical (IHC) staining. Compared with stage I of COAD, the expression of AEN was higher in stage III of COAD (*p* ˂0.01), while the expression of LGALS4 and XDH were lower in the sample of patient with stage III of COAD (*p* ˂0.01) (Figure 9F). These results are in line with the results of our previous analysis.

### Single-cell data analysis

We used single-cell data to further verify the differential expression of the target genes between the cancer and normal tissues. After quality control analysis of the single-cell sequencing data of colorectal cancer tissues and normal tissues, transcriptome gene expression profiles and epithelial cell gene expression profiles were obtained. Comparing the total expression differences of the three target genes in all cells of cancer tissue and normal tissue, the results suggest that: AEN gene expression is higher in cancer tissue than normal tissue, while LGALS4 and XDH gene expression is lower in cancer tissue (Supplementary Figures S1A–C). The expression of target genes in normal intestinal epithelial cells and cancer cells was compared, and the results showed that compared with normal intestinal epithelial cells, the expression of AEN gene was higher in cancer cells, and the expression of LGALS4 and XDH genes was lower in cancer cells (Supplementary Figures S1D–F). These results are consistent with our previous finding that LGALS4 and XDH are protective genes while AEN confers risk.

## Discussion

Colon cancer is the third most common type of cancer, with high morbidity and mortality (Siegel et al., 2019). With the improvement of the cancer treatment, the five-year survival rate of colon cancer patients is approximately 65% (Miller et al., 2019). Although curative resection is a reliable treatment for colon cancer, about a quarter of patients are unable to receive curative treatment due to metastatic cancer, and there is a high risk of recurrence and death after resection (Bray et al., 2018). Therefore, exploration of the effective prognostic markers and therapeutic targets is the key to improve the prognosis of patients with colon cancer.

In recent years, studies have shown that the interaction between tumor cells and immune cells in tumor microenvironment promotes the growth and metastasis of cancer (Lu et al., 2019). The characteristics of immune infiltration in different cancers are closely related to tumor mortality and patient prognosis (Shankaran et al., 2001; Dong et al., 2016; Sahraei et al., 2019). With the continuous exploration of immune mechanism, immunotherapy has provided new methods to difficult-to-treat tumors. However, tumor cells can evolve immune escape mechanisms and produce immunosuppressive microenvironment to promote their growth and metastasis, resulting in reduction of the efficacy of immunotherapy (Riley et al., 2019). Thus, immune-related genes which may affect the tumor immune microenvironment or immune response can be prognostic factors for predicting tumor progression in patients and potential targets for cancer therapy.

In our study, we included samples from 451 patients with colon cancer, identified 7846 differentially expressed genes (DEGs), and screened out 679 immune-related genes among DEGs for further study. Weighted gene co-expression network analysis (WGCNA) was conducted to cluster samples. Grey module of immune-related DEGs was selected to construct a co-expression network. By regression analysis, 6 immune-related DEGs (AEN, F2RL1, NR3C2, PPARGC1A, LGALS4, and XDH) were identified as potential prognostic factors, of which AEN, LGALS4, and XDH were used to construct a risk score model, which can effectively predict overall survival (OS) in colon cancer patients. Besides, immunohistochemistry and single-cell data analysis further verify our model genes. Compared with other research on the prognostic value of tumor microenvironment-related genes in colon cancer, we explored the immune functions and immune escape associated with prognosis (Chen et al., 2021; Guo et al., 2021; Luo et al., 2021). Thus, our study provides a novel and reliable method to evaluate the prognosis of colon cancer patients, a reference for other researchers to explore the immune cells that affect the prognosis, and a new reference method for the prediction of the effect of immunotherapy.

The functional pathways of immune-related DEGs were identified by enrichment analysis. GO enrichment analysis revealed that these DEGs were mainly enriched in humoral immune response, complement activation and lymphocyte-mediated immunity. The immune system is widely believed to help suppress the initiation and development of tumors. However, B cells have been shown to produce antibodies that directly activate growth factor receptors on the tumor cells and promote tumor growth (Lin et al., 2020). A previous study showed that complement activation products are involved in regulation of tumor growth and metastasis (Klos et al., 2013). Besides, lymphocyte-mediated immunity has been used in immunotherapy for cancer patients. Endogenous cytotoxic T cells and exogenous Chimeric Antigen receptors T cells activated by tumor-specific antigens can promote the killing of cancer cells, which is beneficial to the treatment of cancer patients (Leko and Rosenberg, 2020). KEGG pathway analysis showed that immune-related DEGs regulated the development of colon cancer by significantly influencing the cytokine-cytokine receptor interaction, viral protein interaction and neuroactive ligand-receptor interaction pathways. These results suggest that immune-related DEGs play a key role in tumor progression and treatment by participating in related biological functions and signaling pathways, and may become therapeutic targets and prognostic markers for colon cancer patients.

The risk prognostic model we constructed included AEN, LGALS4 and XDH genes, among which AEN was a risk factor unfavorable to prognosis, while LGALS4 and XDH were protective factors favorable to prognosis. AEN (Apoptosis Enhancing Nuclease) is a p53-induced exonuclease involved in cell apoptosis. It has been reported that the expression of AEN increased significantly in peripheral blood lymphocytes of healthy people after exposure to radiation (Turtoi and Schneeweiss, 2009). AEN has been shown to be significantly associated with the prognosis of hepatocellular carcinoma and breast cancer (Zhu et al., 2019; Yin et al., 2021). Several studies demonstrated that LGALS4 which encodes galectin-4 acts as a tumor suppressor to reduce the invasion and metastasis of cancer cells and low expression of LGALS4 was significantly associated with shorter disease-free survival (Belo et al., 2013; Long and Campbell, 2017). XDH (Xanthine dehydrogenase) is not only related to immune reaction, but also participates in the oxidative metabolism of purine. XDH has been indicated in multiple cancers. For instance, low expression of XDH predicted a poor prognosis in patients with breast and serous ovarian cancer (Liu et al., 2018). In addition, the expression level of XDH has been reported to be available in diagnosing colorectal cancer stages (Linder et al., 2009). Combined with the above experimental studies, AEN, LGALS4, and XDH may be related to the progression of colon cancer. Risk scores based on these immune-associated DEGs can also effectively reflect patient outcomes.

Results of immune cell infiltration in colon cancer samples showed significant differences in plasma cells, T cells CD4 memory resting, and macrophages M1 between the high-risk and low-risk groups. Immune cell related function analysis revealed that CCR (chemokine receptor), parainflammation and T-cell co-stimulation were significantly reduced in high-risk groups compared to low-risk groups. Tumor immune microenvironment is one of the major components of tumor microenvironment, consisting of various immune cells infiltrating the tumor (Teh and Aplin, 2019). The interaction between chemokines and CCR located in tumors and stromal cells is involved in the regulation of immune cell infiltration, angiogenesis, and tumor growth (Mollica Poeta et al., 2019). It has been found that inhibition of CCR7 can effectively reduce the number of tumor metastases in colon cancer models (Yu et al., 2008). Parainflammation, a low-grade form of inflammation, is common in cancers that contain p53 mutations, including colon cancer (Aran et al., 2016). Most cancers that respond to aspirin treatment present high parainflammation level (Rothwell et al., 2011). With the study of the molecular mechanism of immunity, increasing number of research have focused on immunotherapy targeting T cell co-signaling, including T cell co-stimulation and co-inhibition (Chen and Flies, 2013). In the T cell co-stimulation pathway, OX40 activation combined with TCR stimulation can promote the differentiation of CD4 and CD8 T cells and improve the cell survival rate (Pourakbari et al., 2021). These results reveal that cancer-related immune cell functions provide potential therapeutic targets for immunotherapy. In addition, correlation analysis demonstrated that T cell exclusion potential was significantly increased in the high-risk group. It is suggested that the poor prognosis of high-risk colon cancer patients may be related to the mechanism of immune escape. Although immunotherapy has brought new advances in tumor treatment, cancer rapidly acquires drug resistance due to tumor immunosuppressive microenvironment and immune escape (Vago and Gojo, 2020). T cell exclusion, as a major part of immune escape, can lead to a lack of T cell infiltration within the tumor and poor immune response against tumor (Lin et al., 2021). A previous study has shown that tumors with T cell exclusion have angiogenic factors with high expression level, resulting in promotion of tumor metastasis (Harlin et al., 2009).

However, there are still some limitations in our study. The prognostic model needs more high-quality and larger sample clinical study to further validate its prognostic value. Biological experiments are required to explore the underlying mechanisms of immune-related prognostic genes in colon cancer.

## Conclusion

In conclusion, our study established a risk model based on immune-related genes to predict overall survival of patients with colon cancer which may become the potential prognostic evaluation method. Besides, we found significant differences in immune cell infiltration, immune cell functions and immune escape potential in low-risk and high-risk groups, which provides a novel direction for exploring potential targets of immunotherapeutic in colon cancer patients.

## Data Availability

The datasets presented in this study can be found in online repositories. The names of the repository/repositories and accession number(s) can be found in the article/Supplementary Material.

## Supplementary material

Supplementary Figure S1 Experimental validation of differential expression of target genes. **(A–C)** Differential expression of AEN, LGALS4 and XDH in all cells of cancer and normal tissues by single-cell sequencing. **(D–F)** The differential expression of AEN, LGALS4 and XDH in normal intestinal epithelial cells and cancer cells was detected by single-cell sequencing.
