# Supplementary figures and images for: Construction of a prognostic assessment model for colon cancer patients based on immune-related genes and exploration of related immune characteristics
Source: Front Cell Dev Biol. 2022 Dec 16;10:993580. doi: 10.3389/fcell.2022.993580 (PMC9800979; doi:10.3389/fcell.2022.993580)

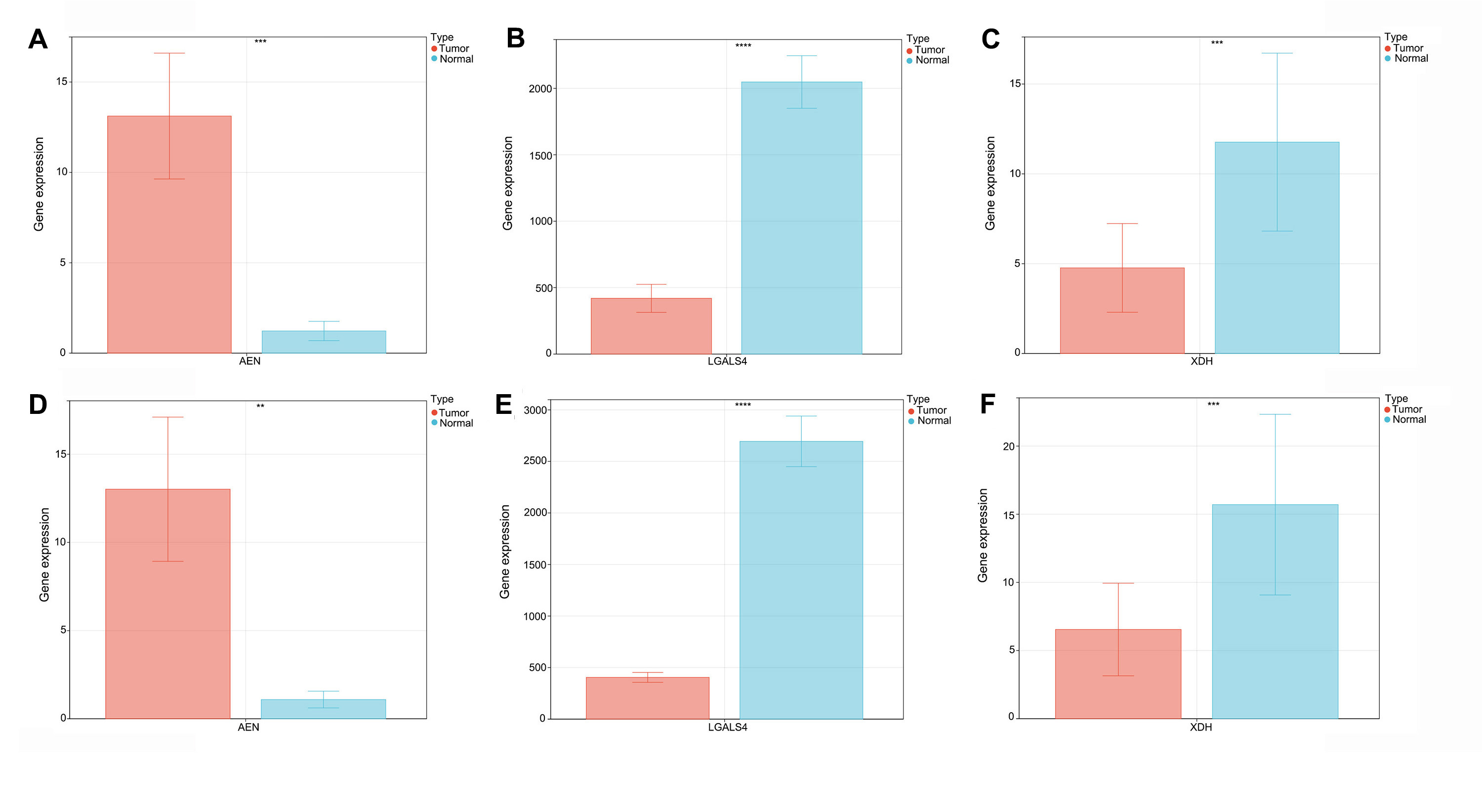

Supplement: Supplementary file 1 [file Image1.jpg]
